# Southern Medical College

**Published:** 1881-10-20

**Authors:** 


*Southern Medical College.*—The introductory address at the opening of the Southern Medical College on the 13th instant was delivered by Prof. Jno. Thad. Johnston, in the presence of the class and an intelligent audience of citizens. The address was an able one and was listened to with marked attention.

